# Causal Relationship between Chronic Hepatitis B and Stroke in East Asians: A Mendelian Randomization Study

**DOI:** 10.3390/jcdd11080247

**Published:** 2024-08-10

**Authors:** Qi Zhang, Cancong Shen, Lei Zhang, Maiqiu Wang

**Affiliations:** 1 School of Information and Electronic Engineering, Zhejiang University of Science and Technology, Hangzhou 310023, China; 222109252030@zust.edu.cn (Q.Z.); leizhang@zust.edu.cn (L.Z.); 2 School of Biological and Chemical Engineering, Zhejiang University of Science and Technology, Hangzhou 310023, China; bazinga_shen@163.com

**Keywords:** chronic hepatitis B, stroke, Mendelian randomization

## Abstract

Both chronic hepatitis B (CHB) and stroke contribute to a high burden of disease in the majority of low- and middle-income countries. Epidemiological studies yield conflicting results on the association between CHB and stroke, and the causal relationship remains inconclusive. This study aimed to assess the causal effects of CHB on stroke and its subtypes in East Asians by Mendelian randomization (MR) analysis. Variants associated with CHB were obtained from a genome-wide association study (GWAS) of Chinese samples as instrumental variables. The summary statistics for stroke in East Asians were derived from the largest published GWAS to date. Two-sample MR analyses were implemented to evaluate the causal effects of CHB on stroke and its subtypes by using the canonical inverse variance weighting method and other supplementary approaches. We observed an association between genetic predisposition to CHB and a decreased risk of large-artery atherosclerotic stroke (odds ratio = 0.872, 95% confidence interval = 0.786–0.967, *p* = 0.010). The causal effects of CHB on other stroke outcomes were not statistically significant. Evidence for heterogeneity and horizontal pleiotropy were not found in our analyses. This study provides genetic evidence for a negative association between CHB and stroke in East Asians, which helps improve our understanding of the etiology of stroke.

## 1. Introduction

Stroke is caused by cerebral ischemia due to vascular obstruction or cerebral hemorrhage and is characterized by sudden onset of neurological deficits. There are more than 10 million incident cases of stroke annually, and the burden attributable to the disease remains high [1]. Stroke is the leading cause of death and disability, accounting for approximately 12% of deaths worldwide [1]. In addition to genetic variations, many risk factors are involved in the development of stroke, such as smoking, hypertension, dyslipidemia, and diabetes [2]. However, the relationship between various plausible exposure factors and stroke remains to be explored.

Infection and chronic inflammation have been shown to play a role in the development of major adverse cardiovascular events [3,4]. For example, hepatitis C virus (HCV) infection is a well-documented risk factor for cardiovascular disease (CVD) and stroke [5,6]. This conclusion cannot be extrapolated for hepatitis B virus (HBV), although there are some similarities in the clinical manifestations of hepatitis caused by the two viral infections. A longitudinal study including 31,943 subjects reported a significantly higher risk of ischemic stroke in HCV-infected patients compared with HBV-infected patients [7]. Epidemiological observations on the association between stroke and chronic hepatitis B (CHB) are controversial. Three prospective studies reported the association of CHB with a reduced risk of ischemic stroke [8,9,10], whereas other studies have found no evidence to support this finding [11,12,13].

The corresponding vaccines can effectively prevent HBV infection, but there were still 316 million people living with CHB in 2019 [14]. The burden of CHB disproportionately affects Africa and East Asia, which is similar to stroke [1]. Therefore, it is worthwhile to determine the association between CHB and stroke. Mendelian randomization (MR) is an emerging statistical method using genetic variants as instrumental variables (IVs) to infer causal relationships between exposure factors and outcomes. Since most of the CHB-associated variants were identified in East Asians and the summary statistics of a genome-wide association study (GWAS) on stroke in East Asians were not accessible, it was not feasible to perform corresponding MR analysis. Very recently, a large-scale GWAS meta-analysis on stroke was conducted across ancestry, and the summary statistics of East Asians have been made public [15]. In this study, we aimed to assess the causal effects of CHB on stroke and its subtypes in East Asians by MR analysis.

## 2. Materials and Methods

### 2.1. Study Design and Data Sources

A two-sample MR study was conducted to assess the causal relationship between genetic susceptibility to CHB and stroke risk in samples of East Asian ancestry. The MR design was based on three main hypotheses: (i) the genetic variants used as IVs were associated with exposure (i.e., CHB); (ii) IVs were not associated with confounders; (iii) IVs influenced the risk of outcome (i.e., stroke and its subtypes) only through changes in the exposure.

Summary statistics of single nucleotide polymorphisms (SNPs) associated with CHB were derived from a GWAS of Chinese samples [16]. The study included 9114 cases and 9257 controls and identified 12 significant SNPs [16]. CHB was defined as the persistent presence of hepatitis B surface antigen and immunoglobulin G antibody to hepatitis B core antigen for at least six months.

We obtained the GWAS summary statistics for stroke from the largest published GWAS to date and exclusively utilized the data from East Asian individuals [15]. The East Asian sample used in our study comprised a total of 27,413 stroke cases and 237,242 controls, consisting of individuals of Japanese, Chinese, and Korean descent [15]. There were five stroke outcomes, including any stroke (AS, 27,413 cases), any ischemic stroke (AIS, 19,032 cases), large-artery atherosclerotic stroke (LAS, 1735 cases), small-vessel stroke (SVS, 5532 cases), and cardioembolic stroke (CES, 926 cases). The LAS, SVS, and CES subtypes were classified according to the Trial of Org 10172 in Acute Stroke Treatment (TOAST) criteria [17]. The full summary statistics of stroke outcomes were downloaded from the GWAS Catalog (GCST90104544-GCST90104548) [18].

### 2.2. Statistical Analyses

Outcome data were extracted for the SNPs identical to the IVs and harmonized with the exposure data prior to MR analyses, which ensured that the effect of each SNP for both data corresponded to the same allele. Three MR methods were used to assess causal effects, including inverse variance weighting (IVW) [19], MR-Egger, and weighted median [20,21]. Canonical MR analyses use independent IVs (i.e., variants not in linkage disequilibrium (LD)) to avoid overcounting effects. To obtain independent IVs, clumping was performed on the condition of LD r^2^ < 0.01 and distance cutoff < 10,000 kb. Eleven of the twelve SNPs are located at the human leukocyte antigen region, and only four SNPs were retained after clumping. It has been shown that the efficiency of MR analysis can be improved by using multiple variants in each gene region when accounting for the correlation between variants [22]. Thus, we additionally employed this approach to conduct MR analyses. In summary, for each pair of exposure and outcome, there were five MR estimates: (i) IVW method using independent SNPs (IVW (canonical)); (ii) IVW method using correlated SNPs (IVW (all SNPs)); (iii) MR-Egger method using independent SNPs (MR-Egger (canonical)); (iv) MR-Egger method using correlated SNPs (MR-Egger (all SNPs)); (v) weighted median method. The IVW (canonical) was the main method to infer causal relationships, while other MR estimates were supplementary evidence.

### 2.3. Sensitivity Analyses

To avoid weak instrument bias, the strength of each IV was assessed by the F-statistic using the formula F = (R^2^ × (N − 2))/(1 − R^2^), where R^2^ referred to the variance explained by the SNP and N was the sample size of exposure data [23]. The association between an IV and exposure was considered sufficiently strong when the F-statistic was greater than 10. The MR-Egger intercept method was used to estimate the likelihood of horizontal pleiotropy [20]. Heterogeneity among SNPs was measured by the Cochran’s Q test [19]. All performed MR analyses and sensitivity analyses were carried out using the TwoSampleMR (version 0.5.6) and MendelianRandomization (version 0.7.0) packages in the R environment (version 4.1.1) [24,25].

## 3. Results

Among 12 SNPs associated with CHB, 4 were independent after LD clumping, namely rs1883832, rs7453920, rs9277535, and rs1419881. The summary statistics of variant rs2853953 were not available in the stroke outcomes, and we excluded that SNP in subsequent MR analyses. All SNPs had an F-statistic greater than 10, indicating no evidence of weak instrument bias. Detailed information on IVs can be found in Table 1.

**Figure 1:**
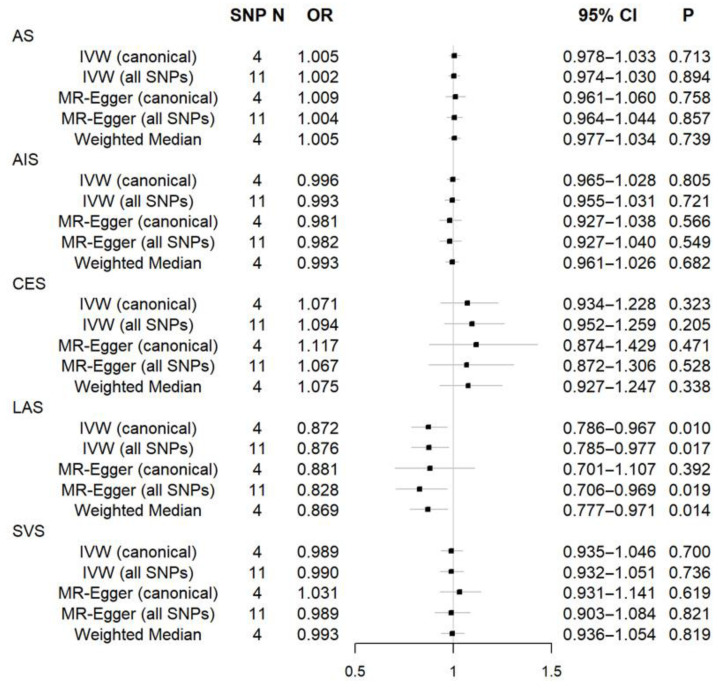
Forest plot for causal effects of chronic hepatitis B on stroke and its subtypes. AS = any stroke, AIS = any ischemic stroke, CES = cardioembolic stroke, LAS = large-artery atherosclerotic stroke, SVS = small-vessel stroke, IVW = inverse variance weighting, SNP = single nucleotide polymorphism, N = number, OR = odds ratio, CI = confidence interval.

All MR results are exhibited in Figure 1. Genetic predisposition to CHB was significantly negatively associated with LAS in the analysis using the IVW (canonical) method. The odds ratio per unit decrease in log odds of CHB was 0.872 (95% confidence interval = 0.786–0.967, *p* = 0.010) for LAS, and the MR results based on IVW (all SNPs), MR-Egger (all SNPs), and weighted median methods were consistent with the IVW (canonical) method. The association was still significant after Bonferroni correction for five stroke outcomes (*p* < 0.05/5 = 0.01). MR results using all SNPs were similar to results using independent SNPs, except for the MR-Egger method in LAS (inconsistent statistical significance) and SVS (opposite effect). In addition to multiple MR methods, we also performed a series of sensitivity analyses to demonstrate the robustness of our results. The MR-Egger intercept test indicated that the association was not driven by directional horizontal pleiotropy (Table 2). Further, no heterogeneity among SNPs was proved by Cochran’s Q test, as shown in Table 2.

## 4. Discussion

We conducted MR analyses to assess the causal effects of CHB susceptibility on stroke and its subtypes. Our results suggest that genetic liability to CHB is associated with a decreased risk of LAS in East Asians, but other associations were not observed in AS, AIS, SVS, and CES.

People with CHB are exposed to high rates of some extrahepatic complications, and systemic vasculitis is one of the well-described manifestations [26]. However, this inflammation and damage in blood vessel walls does not appear to increase the risk of major CVD events in CHB patients [26]. Conversely, several studies have provided evidence that CHB may reduce the risk of developing ischemic stroke [8,9,10]. The association was also supported by a recent meta-analysis involving 83,475 HBV-infected individuals and 593,949 uninfected controls [27]. The liver plays an important role in the metabolism of lipid and glucose. Unexpectedly, CHB is not associated with an increased risk of metabolic syndrome, and most evidence points to a protective effect of chronic HBV infection against metabolic syndrome [28]. Furthermore, individuals with CHB have a lower incidence of hypercholesterolemia and hypertriglyceridemia [29,30,31,32], which are risk factors for atherosclerosis. The decreased coagulation function caused by CHB may also play a protective role against ischemic stroke [33,34]. In contrast to a lower risk of ischemic stroke, the risk of hemorrhagic stroke is increased in HBV-infected individuals, especially in patients with liver dysfunction [8]. Patients with CHB may pay more attention to adopting healthy lifestyles and be less exposed to risk factors of CVD [12]. However, evidence suggests that this protection effect against ischemic stroke is secondary to chronic HBV infection rather than the adoption of an improved lifestyle [8].

Our results only demonstrated the negative causal effect of CHB on LAS, and such an association between CHB and AIS was not observed in this study. LAS accounts for about 30% to 40% of AIS in East Asians [35,36], whereas LAS accounted for only 9.1% of AIS in our analyses. The different proportions of stroke subtypes may be the explanation for why epidemiological observations are inconsistent with our results. LAS is associated with large-artery stenosis or occlusion caused by atherosclerosis. A 17-year follow-up study based on 22,472 samples indicated that CHB was associated with decreased risks of atherosclerosis-related diseases, but the associations were not significant [13]. A meta-analysis including five studies also found no association between CHB and atherosclerosis-associated disease morbidity. On the contrary, CHB was found to be an independent risk factor for carotid plaques and subclinical atherosclerosis in another small-scale prospective study of 201 subjects [37]. In summary, epidemiological evidence for the association between CHB and atherosclerosis is controversial, and the mechanisms underlying the causal effect of CHB on LAS remain to be determined. CHB increases the risk of detrimental complications such as cirrhosis and liver cancer, and antiviral treatment for HBV is necessary. It cannot be excluded that the protective effect was mediated by antiviral treatment, but relevant research was absent. It is worth considering that if the direct protective effect is from the CHB status, will the antiviral treatment weaken the protective effect? Considering that the mechanisms we discussed were inferred through epidemiological surveys and the observational results were susceptible to confounding factors, further biological function experiments are warranted in the future.

Stroke prevalence and proportions of subtypes vary by ethnicity [1,38]. For example, Asians have a significantly higher incidence of intracranial atherosclerosis than extracranial atherosclerosis [38]. Studies exploring the causal relationship between various exposure factors and stroke in East Asians are very limited. As a common disease in East Asia, CHB was investigated for the first time in our study for its causality with stroke. Multiple MR methods and a series of sensitivity analyses have been performed to verify the robustness of our results. In addition, our study also has some limitations. First, the case sample size for each ischemic stroke subtype was relatively small. In addition, it was noteworthy that the proportion of cases without TOAST classification was disproportionately high in AIS. The cases in the study were derived from 15 cohorts, only 3 of which had TOAST classification data. Among the three cohorts, the TOAST classification proportion in the largest cohort differed greatly from epidemiological studies, with a subtype distribution of 1331 LAS cases, 4915 SVS cases, and 758 CES cases in 17651 AIS cases [15]. Therefore, heterogeneity among stroke cohorts may be a potential factor affecting the results. Second, the causality found in this study is undetermined in other populations. A very recent MR study based on Europeans found that CHB was associated with an increased risk of atherosclerosis and coronary heart disease and a reduced risk of AIS [39]. Two potential explanations account for the different effects of CHB on AIS between the study and our result. The first is that the effect of CHB on stroke is inconsistent across ethnicities [40]. The second explanation involves the data itself. Specifically, even if CHB is indeed negatively associated with AIS, the quality of the data was not sufficient to support the finding. The protective effect of CHB against AIS was attenuated by the low proportion of LAS in AIS.

## 5. Conclusions

This study provides genetic evidence that CHB is associated with a decreased risk of LAS in East Asians. No association was observed between CHB and AS, AIS, CES, and SVS. Further investigations into the mechanism of such a protective effect are warranted to reduce LAS morbidity and mortality.

## Figures and Tables

**Table 1:** Characteristics of instrumental variables.

SNP	Chr	Position	EAF	EA	OA	Beta	SE	*p*	F
rs12614	6	31946402	0.92	C	T	0.637	0.053	1.28 × 10^−34^	144.35
rs422951	6	32220606	0.81	T	C	0.239	0.030	5.33 × 10^−16^	63.25
rs378352	6	33007157	0.39	A	G	0.231	0.022	1.04 × 10^−23^	106.64
rs1883832	20	46118343	0.37	T	C	0.174	0.023	2.95 × 10^−15^	54.77
rs3130542	6	31264334	0.16	A	G	0.157	0.031	8.66 × 10^−7^	26.38
rs652888	6	31883457	0.24	G	A	0.131	0.024	9.92 × 10^−7^	28.03
rs2856718	6	32702478	0.55	T	C	0.247	0.022	7.35 × 10^−28^	125.58
rs7453920	6	32762235	0.88	G	A	0.693	0.045	1.28 × 10^−60^	238.51
rs3077	6	33065245	0.66	G	A	0.371	0.023	1.15 × 10^−53^	265.25
rs9277535	6	33087084	0.58	G	A	0.419	0.024	9.84 × 10^−71^	316.94
rs2853953 *	6	31267728	0.90	G	A	0.385	0.042	5.06 × 10^−20^	85.14
rs1419881	6	31162816	0.55	A	G	0.113	0.023	2.88 × 10^−7^	25.15

SNP, single nucleotide polymorphism; Chr, chromosome; EAF, effect allele frequency; EA, effect allele; OA, other allele; SE, standard error. * Not available in outcome data.

**Table 2:** Results of pleiotropy and heterogeneity tests.

Outcome	4 SNPs		11 SNPs	
	Horizontal Pleiotropy Test	Heterogeneity Test	Horizontal Pleiotropy Test	Heterogeneity Test
	Intercept (*p*)	Q (*p*)	Intercept (*p*)	Q (*p*)
AS	−0.002 (0.875)	0.799 (0.850)	−0.001 (0.903)	6.285 (0.791)
AIS	0.007 (0.581)	0.775 (0.855)	0.004 (0.616)	14.461 (0.153)
CES	−0.019 (0.728)	1.358 (0.715)	0.010 (0.734)	4.425 (0.926)
LAS	−0.004 (0.923)	3.148 (0.369)	0.023 (0.331)	11.203 (0.342)
SVS	−0.019 (0.439)	1.015 (0.798)	0.000 (0.995)	10.855 (0.369)

SNP, single nucleotide polymorphism; AS, any stroke; AIS, any ischemic stroke; CES, cardioembolic stroke; LAS, large-artery atherosclerotic stroke; SVS = small-vessel stroke.

## Data Availability

The datasets generated during and/or analyzed during the current study are publicly available.
